# Long-term outcome and complications of acute correction of canine antebrachial deformities with patient-specific three-dimensional printed osteotomy and reduction guides in 15 dogs

**DOI:** 10.3389/fvets.2025.1533805

**Published:** 2025-10-09

**Authors:** Emmanouil Tzimtzimis, Scott Rutherford

**Affiliations:** Frank. Pet Surgeons, Leeds, United Kingdom

**Keywords:** 3d-printed guides, antebrachial, corrective osteotomy, outcome, complications

## Abstract

**Objective:**

To describe the owner-perceived long-term outcome and complications following acute correction of antebrachial deformities in dogs using patient-specific, three-dimensional (3D)-printed osteotomy and reduction guides and internal fixation.

**Methods:**

Retrospective study of 15 dogs (20 limbs). Medical records of dogs with antebrachial deformities corrected using patient-specific 3D-printed osteotomy/reduction guides and internal fixation with bone plates and a minimum of 1 year of owner follow-up were reviewed. Data collected included patient and surgical details, perioperative, short-, mid-, and long-term outcomes, as well as complications. Pre-surgical planning involved Computer-Aided Design (CAD) subjective segment orientation, from which patient-specific osteotomy and reduction guides were created.

**Results:**

All but one (19/20) limb had full function, and one limb had acceptable function at a mean owner follow-up time of 23 months. The mean long-term Liverpool Osteoarthritis in Dogs (LOAD) and Canine Orthopedic Index (COI) scores were 4.4 and 3.5, respectively. The only minor complication was the breakage of the ulnar plate (1 limb). There were two types of major (I) complications: surgical site infection (3/20 limbs, 15%) and implant-related soft tissue irritation (4/20 limbs, 20%), both of which were treated successfully with implant removal. There were no intraoperative, major (II) or catastrophic complications.

**Clinical significance:**

The acute correction of canine antebrachial deformities aided by patient-specific, 3D-printed osteotomy and reduction guides resulted in excellent long-term outcomes. Complications encountered were surgical site infection and implant-related soft tissue irritation. All complications were successfully treated.

## 1 Introduction

Antebrachial deformities in dogs most commonly result from disturbances of the distal physis of the ulna and/or radius. They may cause lameness and adjacent joint problems, such as incongruency, instability, subluxation and osteoarthritis (1–4). The deformities are complex and may be challenging to assess and treat successfully. In recent years, there has been an increasing effort to accurately assess antebrachial deformities, particularly radial torsion, to improve surgical planning and correction (5–7).

The Center of Rotation of Angulation (CORA) methodology is widely adopted to describe limb deformities (8), and preoperative two-dimensional (2D) geometrical CORA calculations have been described to plan corrective osteotomies in all types of deformities (9–11). However, when there are multiplanar deformities, as is often the case with antebrachial deformities, two-dimensional assessment becomes inherently inaccurate, including two-dimensional assessment on a three-dimensional dataset (12). Radial torsion has been shown to interfere with accurate measurement of angular deformity (6) and may pose a significant challenge in intraoperative realignment of the radius (11, 13, 14). Objectively deriving three-dimensional angular values is complex, especially in a clinical setting, and an alternative approach is subjective segment orientation. This is a 3D assessment technique where, in Computer-Aided Design (CAD) software, subjective visual assessment of the optimal proximal and distal orientation of the bone in all planes is made, and the ostectomy (or ostectomies) required are calculated. This has previously been reported for the planning of antebrachial deformity correction (15, 16).

In addition to the challenges of defining the deformity and assessing the ostectomy (or ostectomies) required, surgical execution of pre-surgical planning can be challenging, and 3D-printed bone models have been created for surgical rehearsal (13, 14, 17, 18). More recently, small studies have used patient-specific osteotomy guides with or without reduction guides, yielding favorable results (15, 16, 19–21). The accuracy of 3D-printed patient-specific osteotomy and reduction guides, as well as drill guides, has been investigated in a small number of cases with positive results (21–23). Previous studies have included small numbers of dogs, with the majority having relatively short follow-up, and the complications have not been fully assessed.

The primary objective of this study was to describe the owner-perceived long-term outcomes following acute correction of antebrachial deformities in a large cohort of dogs, as assessed by subjective segment orientation and aided by patient-specific, 3D-printed osteotomy and reduction guides. A second aim of the study was to report and classify complications encountered using widely accepted criteria.

## 2 Materials and methods

### 2.1 Animals

Medical records of consecutive dogs presented to an orthopedic referral hospital for unilateral or bilateral antebrachial deformities between 2018 and 2021 were reviewed. The inclusion criteria were: diagnosis of angular and torsional deformity of one or both antebrachii with associated forelimb lameness, surgical correction with the aid of 3D printed patient-specific guides for osteotomy and reduction, plate osteosynthesis, a minimum of one follow-up radiographic study, and a minimum of 1 year of owners’ follow-up.

### 2.2 Patient and surgical data collection

The following information was collected for each dog: breed, sex and neuter status, body weight (kg), age at the time of surgery (months), limb or limbs affected, limb operated on (bilateral deformities constituted separate entries), presumed etiology of the deformity, chronicity (days) and severity (mild, moderate or severe) of lameness and concurrent orthopedic abnormalities relating to the deformity. The surgical data collected for each dog included: the number of osteotomies or ostectomies required for the radius and ulna, implants used for internal fixation, intraoperative complications, and postoperative medication and exercise regimen. Short-term outcome was assessed by veterinary follow-up, and long-term outcome was assessed by owner telephone follow-up following the consensus study on reporting of subjective outcome of orthopedic procedures (24). Complications were classified as Minor, Major (I), Major (II), and Catastrophic according to the same consensus study (24). Bone healing at the osteotomy or ostectomy site was assessed with orthogonal radiographs of the affected limbs, including the adjacent joints. The timing of each subsequent follow-up was recorded (in weeks after postoperation) along with the function, complications and radiographic findings, as for the first follow-up. The outcome was obtained at least 12 months after the corrective surgery. It was based on the owner’s assessment of the function of the affected limb (24) and two separate owner questionnaires validated for use in dogs with osteoarthritis [“Liverpool Osteoarthritis in Dogs (LOAD)” and “Canine Orthopedic Index (COI)”]. Descriptive statistics were calculated and presented as mean [range, standard deviation (SD)] for all continuous patient variables.

### 2.3 Preoperative planning

All cases underwent preoperative CT imaging of both forelimbs, with the patient in sternal recumbency and forelimbs extended, using a 16-slice multidetector scanner (GE Revolution ACT 16 slice) under general anesthesia with a standard anesthetic protocol. The CT was set at 120 kVp and 30 mAs, with slice thicknesses of 0.625 or 1.25 mm. The images were assessed in surface rendering for suitability.

**Figure 1:**
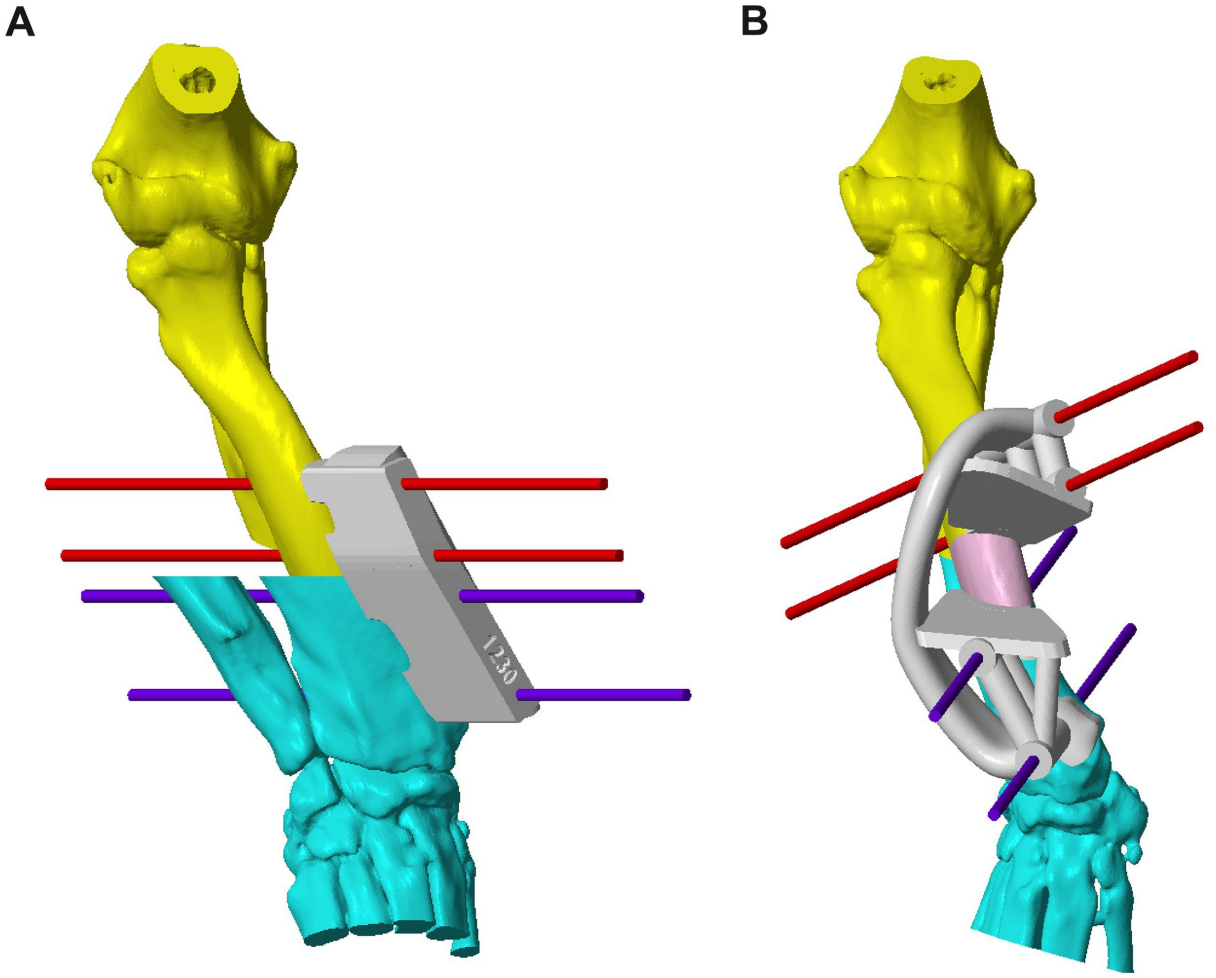
**(A)** CAD image of the right antebrachium of Case 5 with a complex deformity, with a patient-specific osteotomy guide and virtual fixation pins in place. The planes of ostectomy have been calculated based on the subjective segment orientation. **(B)** CAD image of the corrected deformity with a patient-specific reduction guide held in place with the previously inserted pins. An ulna osteotomy has been performed prior to the radial ostectomy. CAD, Computer-Aided Design.

**Figure 2:**
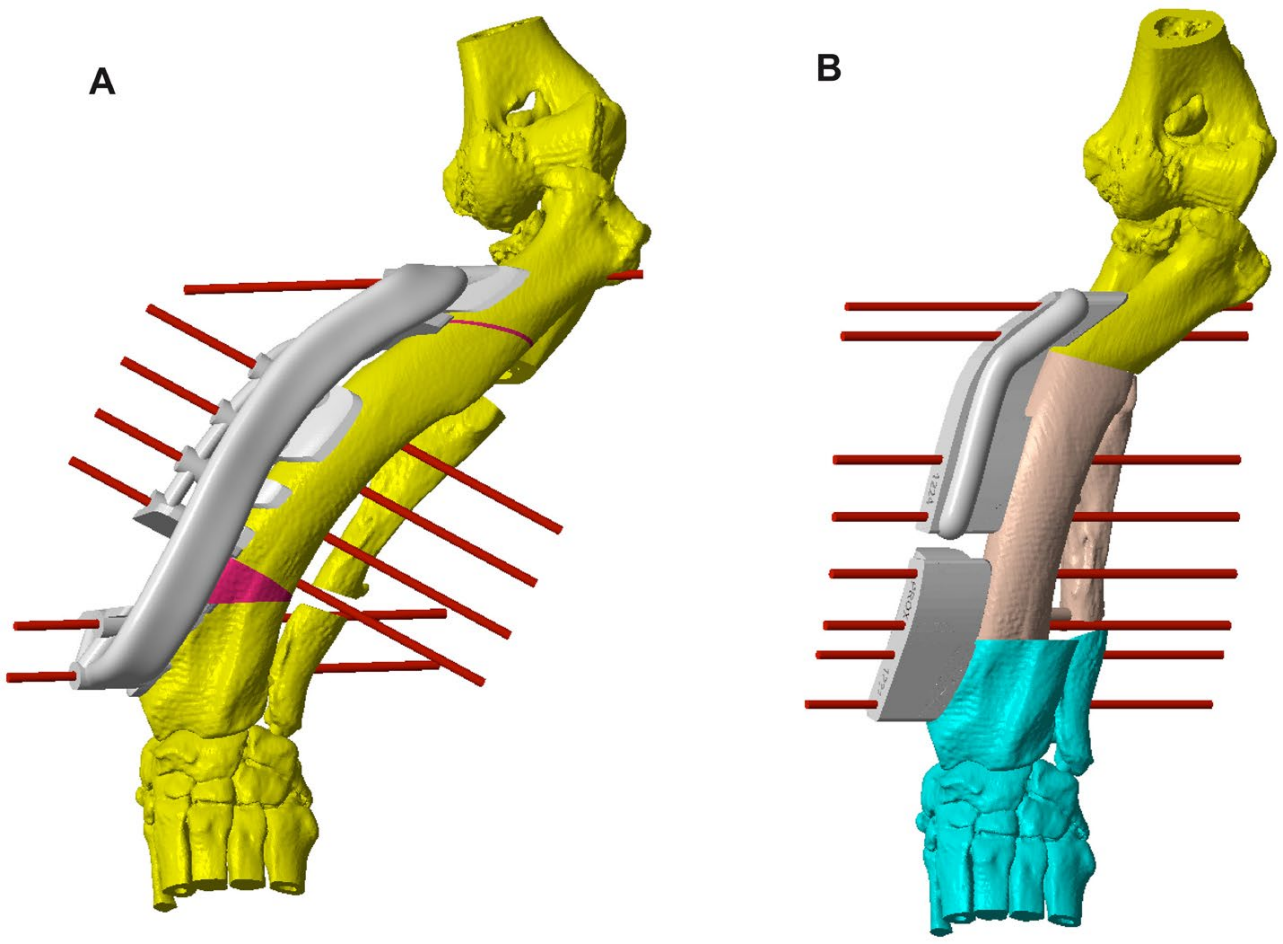
**(A)** CAD image of the left antebrachium of Case 10 with a more severe deformity than that discussed in Figure 1, with a patient-specific osteotomy guide and virtual fixation pins on the radius to allow for the first (distal) radial ostectomy. The first cut of the second (proximal) radial ostectomy is also shown, for which a separate guide was used. A virtual double ulnar osteotomy has been performed prior to the radial correction; **(B)** CAD image of the corrected deformity with a system of two patient-specific reduction guides held in place with virtual fixation pins. CAD: Computer-Aided Design.

The images were exported as stereolithography (STL) files to a CAD software (Netfabb Professional, Netfabb GmbH, Parsberg, Germany) and processed by a third-party company (Vet3D, Cumbria, United Kingdom). Since the majority of cases had developmental bilateral deformities, it was not possible to use a mirrored normal contralateral limb as a 3D template for correction planning in CAD (16). Virtual surgical planning was therefore performed using subjective visual assessment of optimal proximal and distal segment orientation in all planes (subjective segment orientation) (Figures 1, 2) (15).

Determining the optimal ostectomy level was rarely straightforward due to the typical complexity of the overall deformity. The majority had two frontal plane CORAs, and in the sagittal plane, generalized increased procurvatum without a well-defined point of maximal deformity. Frequently, several different ostectomy levels were trialed, and the most visually acceptable was selected based on reduced radial conformation in all planes, ostectomy segment size, and residual translation deformity. Typically, an ostectomy parallel to the distal radial articular surface was made close to the point of maximal deformity in the frontal plane. Since the majority of the deformities reported here were sigmoid in the frontal plane, some residual medial translational deformity after a single-level ostectomy was unavoidable. When the magnitude of this translation was subjectively considered likely to significantly affect the clinical outcome, a double-level correction was planned. For each case, a patient-specific osteotomy and reduction guide system was designed to facilitate the planned correction. The principles of guide system design were identical to those previously described for distal femoral osteotomy (Figures 1, 2) (22).

Stereolithography files of the osteotomy and reduction guides, as well as two bone models (preoperative and postoperative), were exported to Formlabs software (Formlabs, Somerville, MA, United States); they were then prepared for printing and finally exported to a Form 2 or 3 printer (depending on the date and printing material). Pre- and postoperative bone models were printed in high-temperature and white methacrylate photopolymer resin, respectively. The guides were printed using Formlabs Dental SG resin or BioMed Amber resin, depending on the date. These are certified as autoclavable and biocompatible [International Organization for Standardization (EN ISO) 10,993–1, 5, 10, and 11]. All guides and models were cleaned and ultraviolet (UV)-cured according to the manufacturer’s instructions. The postoperative bone models can be used to precontour the implants, which is particularly useful for inexperienced surgeons. No implants were pre-contoured in this case series.

### 2.4 Surgical technique and postoperative care

Dogs were anesthetized with a standard anesthetic protocol. A caudolateral approach to the mid-distal ulnar diaphysis was performed, followed by a free-hand single or double osteotomy of the ulna to facilitate subsequent realignment of the radius. The decision whether to perform a single or double osteotomy was subjective and based on the degree of radial correction required and anticipated soft tissue tension. A craniomedial approach to the radius was then performed with extensive dissection of soft tissues over the area of the bone with the planned footprint of the osteotomy guide The osteotomy guides were applied on the craniomedial aspect of the diaphysis, manipulated to achieve an exact match with the aid of the 3D-printed bone model and secured with 1.4 or 1.6 mm negative profile- threaded pins (Veterinary Instrumentation, Sheffield, United Kingdom), then a single or double closing wedge ostectomy was performed. The segments of the cutting guide were removed, leaving the pins in place, and the reduction guide was applied. Once the desired multiplanar alignment was achieved, a locking fixation system was selected, contoured and applied.

**Figure 3:**
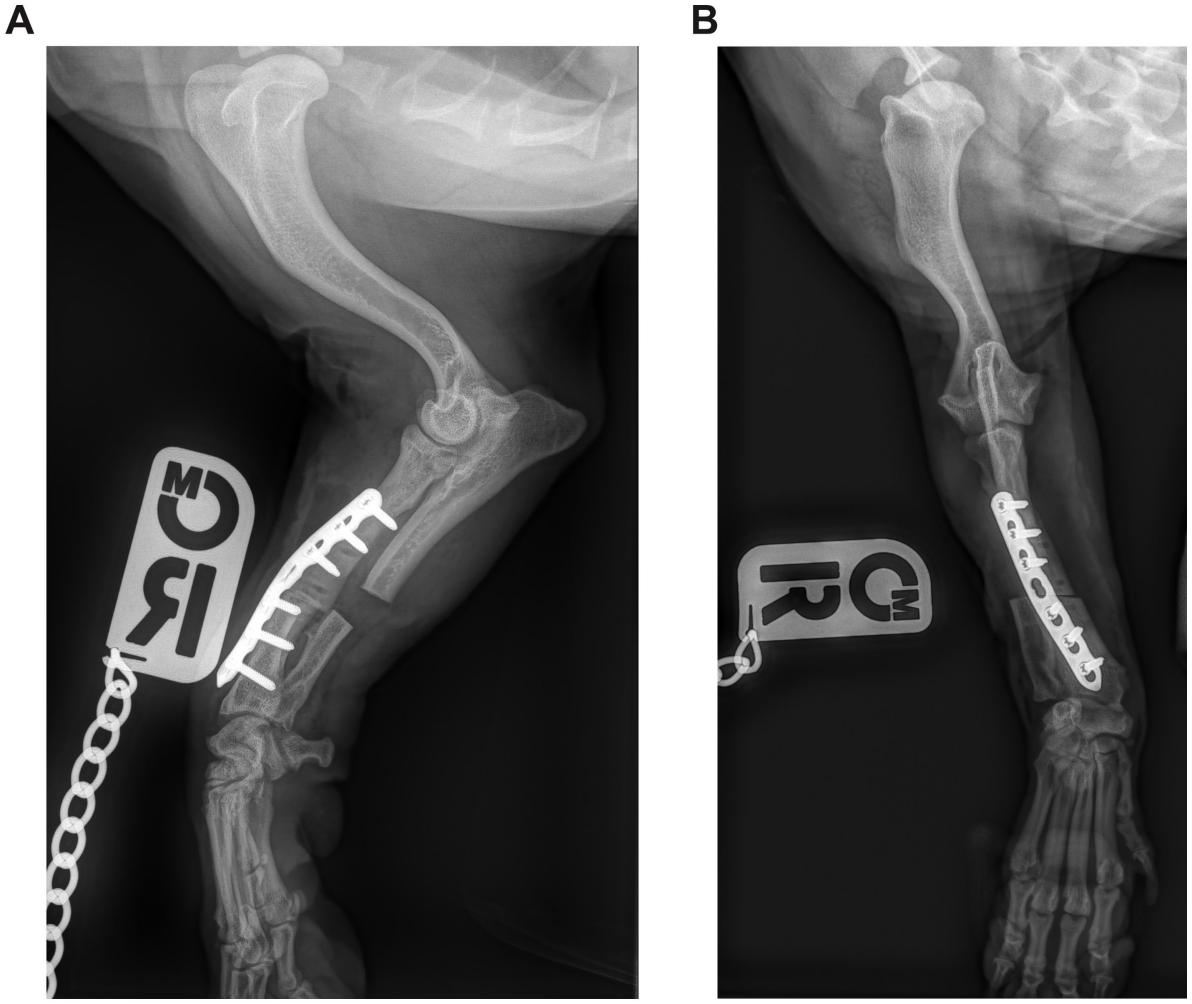
**(A)** Postoperative mediolateral and **(B)** craniocaudal radiographs of the antebrachium of the dog from Figure 1. The ostectomy has been fixed with a single Locking Compression Plate.

**Figure 4:**
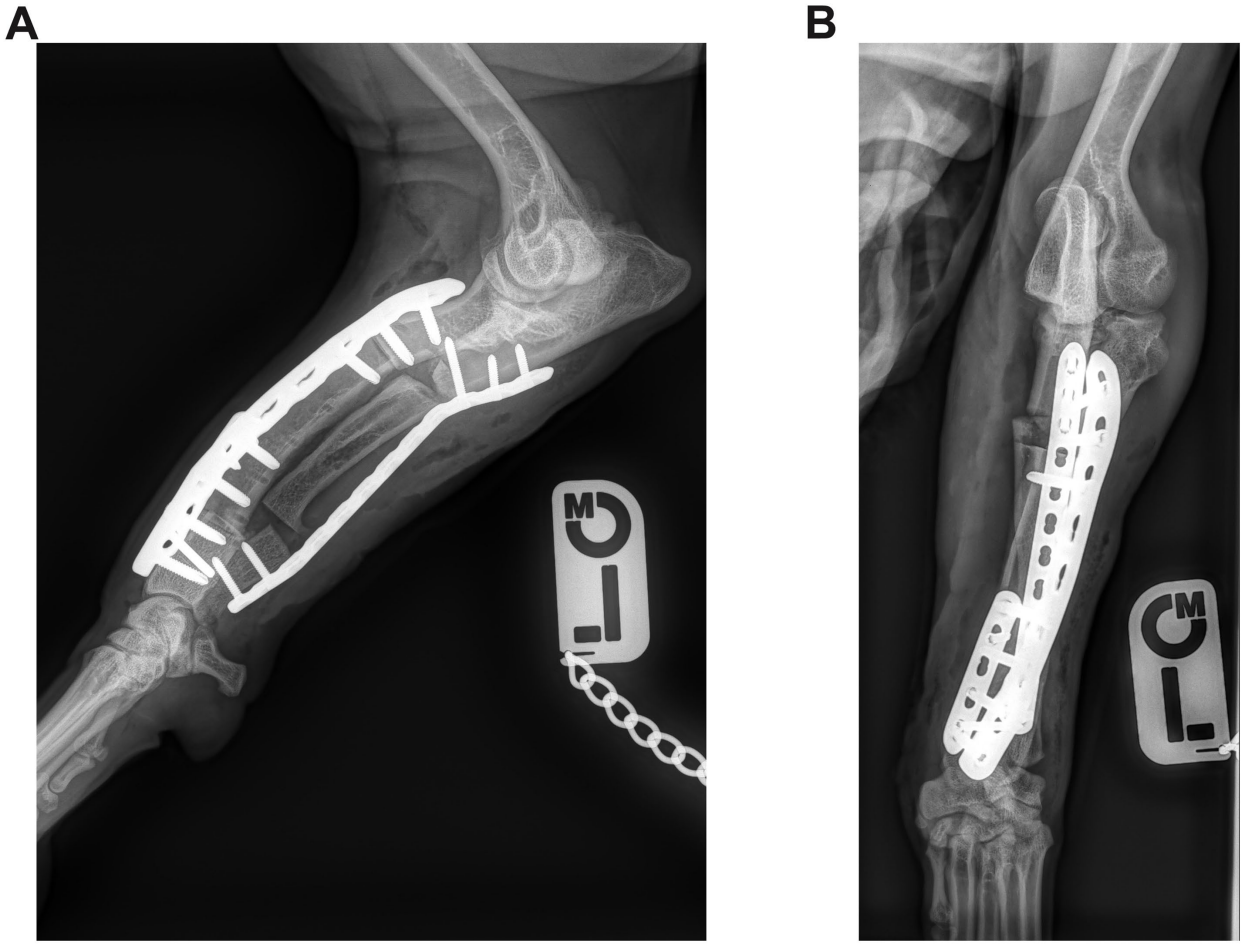
**(A)** Postoperative mediolateral and **(B)** craniocaudal radiographs of the antebrachium of the dog from Figure 2. The radial ostectomies have been fixed with two Locking Compression Plates (LCP), and the ulnar osteotomies have been fixed with a single LCP.

Immediately after surgery, the axial and rotational alignment of the antebrachium was grossly inspected, and orthogonal radiographs were performed to assess the adjacent joints’ orientation and implant position (Figures 3, 4). No objective measurements of antebrachial alignment were recorded. A light bandage was applied to the limb just distal to the elbow to control soft tissue swelling for 24 h. All patients were hospitalized for at least 24 h and received methadone analgesia based on the Short Form of the Glasgow Composite Measure Pain Scale (0.1–0.2 mg/kg every 2–4 h if the score was 6/24 or above).

Patients were discharged with advice to restrict exercise until the first follow-up appointment and to take oral non-steroidal anti-inflammatory medication for at least 2 weeks. Follow-up radiographs of the affected antebrachium were obtained in 6–8 weeks postoperatively to document bone healing at the ostectomy site. Subsequent appointments, with or without radiographs, were scheduled at the discretion of the surgeon.

## 3 Results

Fifteen dogs were identified that satisfied the inclusion criteria (20 limbs), and no dogs were excluded from the study. Thirteen of them had bilateral antebrachial deformities, and five of them underwent bilateral corrective ostectomy. The mean age at presentation was 18.6 months (range, 9–46 months, SD ± 10.5 months). The mean body weight was 16.5 kg (range, 5.7–31.5 kg, SD ± 9.1 kg). Of the dogs that underwent bilateral surgery, one had single-stage corrective ostectomies, one underwent contralateral limb correction within 24 h, and three dogs underwent contralateral limb surgery 8, 11, and 21 months after the first procedure.

### 3.1 Historical and clinical findings

In two dogs, the deformity was suspected to be due to previous physeal trauma (Table 1; Cases 1 and 2). One dog had a distal ulnar retained cartilaginous core (Case 12). In the remaining 12 dogs, the deformity was suspected to be secondary to chondrodystrophy. The mean duration of lameness was 90 days (range, 8–349 days, SD ± 79 days). There was no concurrent neurological disease in any of the dogs. The signalment, presenting clinical signs and concurrent orthopedic abnormalities of each dog are reported in Table 1.

**Table 1:** Signalment, presenting clinical signs and concurrent orthopedic abnormalities relating to antebrachial deformities of 15 dogs that underwent corrective antebrachial ostectomies.

Case	Breed	Age (months)	Body Weight (kg)	Sex (male, female)	Limbs affected (left, right)	Lameness duration (days)	Lameness severity	Carpal instability	Elbow incongruity	Elbow/Carpal OA (E/C)	Concurrent orthopedic problems
1	Lurcher	12	20.3	M	L, R	59	Moderate	None	None	None	None
2	Glen of Imaal Terrier	14	13.5	FN	R	180	Mild	None	None	E	Left forelimb chondrodystrophy
3	Cross	18	11.8	MN	L, R	134	Moderate	Mild	R H-U	E	None
4	Shih Tzu	46	6.5	MN	L, R	90	Moderate	Moderate	None	None	Mild right antebrachial deformity
5	Shih Tzu	21	11.3	MN	L	98	Moderate	Mild	H-U	None	None
		42	12.6		R	35	Moderate	Mild	H-U	None	
6	Cross	23	31.5	M	L, R	24	Moderate	Moderate	None	None	None
7	Jack Russell Terrier	36	10	FN	L, R	45	Moderate	None	None	None	None
8	Basset Hound	12	23.7	M	L, R	172	Moderate	None	L, R	None	Left ununited anconeal process treated with a lag screw and PUO 5 months prior to correction
9	Cross	25	11.9	M	L	58	Moderate	None	None	None	None
		36	13.3		R	8	Mild	None	None	None	
10	Cross	10	27.5	F	R	71	Severe	None	R with radial head subluxation	None	Bilateral DUO 5.5 months prior to the first correction
		18	31.7		L	132	Severe	None	L with radial head subluxation	None	
11	Labrador Retriever	16	25	M	L, R	349	Moderate	Mild	L with radial head subluxation	None	Bilateral DUO and left epiphysiodesis 11 months prior to surgery, bilateral mild medial coronoid disease
12	Labrador Retriever	9	31.5	M	L	111	Mild	None	H-U and radial head subluxation	None	Left distal ulna retained cartilaginous core and metaphyseal osteopathy, DUO 5 months prior to correction
13	Cross	13	7.6	M	L, R	60	Moderate	None	None	None	None
14	Lhasa Apso	15	9.8	MN	L, R	89	Moderate	Mild	None	None	None
15	Jack Russell Terrier	9	5.7	M	L, R	29	Moderate	None	None	None	None

N, Neutered; OA, Osteoarthritis; H-U, Humero-ulnar; PUO, Proximal Ulnar Osteotomy; DUO, Distal Ulnar ostectomy.

### 3.2 Surgical variables

Twelve limbs (9 dogs) had a combination of a single radial ostectomy and single ulnar osteotomy, five limbs (4 dogs) had single radial ostectomy and double ulnar ostectomy, and three limbs (2 dogs) had double radial ostectomy and double ulnar osteotomies. The locking systems used were the String of Pearls (SOP, Orthomed, Huddersfield, United Kingdom), Locking Compression Plate (LCP, DePuy Synthes, NJ, United States) or Stacked Locking Plate (Veterinary Instrumentation, Sheffield, United Kingdom). The surgical variables of all cases are presented in Table 2. There were no intraoperative complications in any of the cases. A European College of Veterinary Surgeons (ECVS) boarded surgeon performed 18/20 procedures, and two non-specialists performed the other two procedures.

**Table 2:** Surgical variables and complications of 15 dogs that underwent corrective antebrachial ostectomies.

Case	Side affected	Correction (radial ostectomy/ulnar ostectomy)	Implants (position, length [holes], size and type of plate, screws proximal and distal, working length [screw holes])	Complications
1	Left	Single/single	Cranial 10 h 2.7 mm SOP, 4, 4, 2 Medial 7 h 2.7 mm SOP, 3, 3, 1	None
	Right	Single/single	Cranial 10 h 2.7 mm SOP, 4, 4, 2 Medial 7 h 2.7 mm SOP, 3, 3, 1	None
2	Right	Single/single	Cranial 6 h 2.7 mm SOP, 3, 2, 1 Medial 4 h 2.7 mm SOP, 2, 2, 0	Surgical site infection
3	Right	Single/single	Cranial 7 h 2.7 mm SOP, 3, 3, 1 Medial 6 h 2.7 mm SOP, 2, 2, 2	None
4	Left	Single/single	Cranial 7 h 2.0 mm low profile (LP) SOP, 3, 3, 1	None
5	Left	Single/single	Cranial 7 h 2.4 mm VI Stacked Locking Plate, 3, 3, 1	None
	Right	Single/single	Cranial 7 h 2.4 mm LCP, 3, 3, 1	None
6	Left	Single/single	Cranial 8 h 3.5 mm SOP, 3, 3, 2 Medial 8 h 2.7 mm SOP, 3, 3, 2	None
	Right	Single/single	Cranial 8 h 3.5 mm SOP, 3, 3, 2 Medial 10 h 2.7 mm SOP, 3, 3, 4	None
7	Left	Single/double	Cranial 7 h 2.4 mm VI Stacked Locking Plate, 3, 3, 1	None
8	Left	Single/single	Cranial 7 h 2.7 mm SOP, 3, 3, 1 Medial 7 h 2.7 mm SOP, 3, 3, 1	Surgical site infection
9	Left	Single/double	Cranial 7 h 2.0 mm LP-SOP, 3, 3, 1 Medial 5 h 2.0 mm SOP, 2, 2, 1	Implant-related soft tissue irritation
	Right	Single/double	Cranial 6 h 2.4 mm LCP, 3, 3, 0	None
10	Right	Double/double	Cranial-distal 7 h 2.7 mm LCP-T, 2, 3, 2 Cranial-proximal 6 h 2.7 mm SOP, 2, 3, 1 Medial 9 h 3.5 mm SOP, 2, 2, 2, 1, 2	Implant-related soft tissue irritation
	Left	Double/double	Cranial 9 h 3.5 mm LCP, 3, 4, 2 Medial 5 h 2.7 mm LCP, 2, 1, 2 Caudal ulnar 12 h 2.7 mm LCP, 3, 2, 7	Implant-related soft tissue irritation, breakage of the ulnar plate
11	Left	Single/single	Cranial-medial 10 h 2.7 mm SOP, 3, 2, 4 Cranial-lateral 9 h 2.4 mm LCP-T, 4, 3, 1	None
12	Left	Double/double	Cranial-distal 6 h 2.7 mm LCP-T, 2, 2, 1 Cranial-proximal 6 h 2.7 mm LCP, 3, 2, 1 Medial 11 h 3.5 mm SOP, 3, 2, 2, 1, 3	Implant-related soft tissue irritation
13	Right	Single/double	Cranial 6 h 2.4 mm LCP, 3, 3, 0	None
14	Left	Single/double	Cranial 6 h 2.4 mm LCP, 3, 3, 0	None
15	Right	Single/single	Cranial 6 h 2.0 mm LCP, 3, 3, 0	Surgical site infection

SOP, String of Pearls Plate; VI, Veterinary Instrumentation; LCP, Locking Compression Plate; LCP-T, Locking Compression T-Plate.

### 3.3 Perioperative, short-term, and mid-term outcomes

Patients were re-examined by the surgeon who performed the procedure, in a mean time of 6.8 weeks (range, 4–11 weeks, SD ± 1.6 weeks) after the procedure, and had at least one follow-up radiographic study of the affected antebrachium. Nine dogs (12 limbs) underwent multiple follow-up examinations with a mean time of the last examination of 28.3 weeks (range, 14–57 weeks, SD ± 14.5 weeks). At the time of the first re-examination, the limb function was acceptable in 18/20 cases. Follow-up radiographs revealed radiographic union at the radial ostectomy site(s) in all cases. There was evidence of progressive or complete healing in 12/20 ulnar osteotomies in total. Among the limbs with a single ulnar osteotomy, 4/12 had delayed/non-union. Among the limbs with double ulnar osteotomies, 4/8 had complete healing at one site and delayed/non-union at the other site.

### 3.4 Complications

There was one minor complication (1/20, 5%), involving the breakage of the ulnar plate, which was discovered during radiography at the first follow-up examination (8 weeks) for Case 10. There were two types of major (I) complications: surgical site infection (SSI) [3/20 limbs, 15%] and implant-related soft tissue irritation (4/20 limbs, 20%). Surgical site infection was diagnosed based on clinical signs of pain, swelling and purulent discharge with response to broad-spectrum antibiotic treatment (cephalexin 10–25 mg/kg or amoxycillin–clavulanic acid 12.5-–25 mg/kg PO BID for 2–6 weeks). The infection resolved after implant removal in all cases. One of these dogs underwent staged implant removal 5 weeks apart (Case 2), while the other two dogs underwent single-stage implant removal (Cases 8 and 15). Implant-related soft tissue irritation was diagnosed based on clinical signs (persistent lameness, decreased carpal flexion and pain on carpal flexion) and response to removal of the implants. One case underwent partial implant removal from both limbs (Case 10), one case underwent single-stage complete implant removal (Case 9), and one case underwent staged complete implant removal 9 weeks apart (Case 12). There were no major (II) or catastrophic complications. Complications are summarized in Table 2.

### 3.5 Long-term outcome

**Figure 5:**
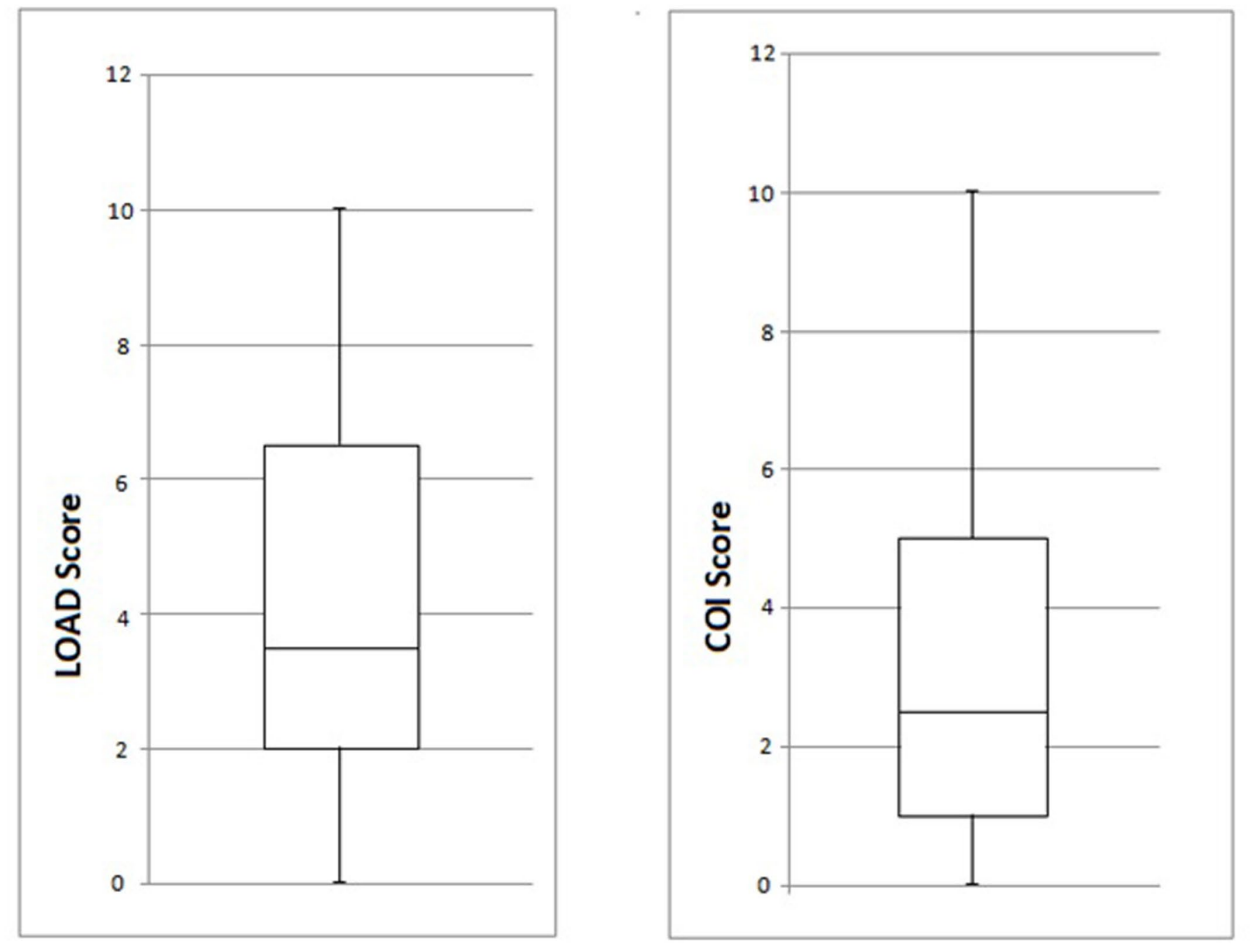
Box and whisker diagrams of LOAD and COI scores (min, Q1, median, Q3 and max) for the 20 limbs of the study at the long-term owner follow-up. LOAD, Liverpool Osteoarthritis in Dogs; COI, Canine Orthopedic Index.

Owners were contacted via telephone and were asked to answer a questionnaire that allowed forelimb(s) function to be assessed using the criteria described by Cook et al. (24), in a mean of 23 months (range, 12–43 months, SD ± 10.9 months) and by completion of the LOAD and COI questionnaires. There was full function of 19/20 limbs. Case 10 had a recurrence of an intermittent right thoracic limb lameness 14 months post-surgery. On follow-up orthopedic examinations, pain was localized to the elbow joint. This lameness was partially responsive to carprofen, and the dog returned to acceptable function. The mean LOAD score at the owners’ follow-up was 4.4 (range, 0–10, SD ± 3.3), and the mean COI score was 3.5 (range, 0–10, SD ± 3.3). Of the dogs that underwent bilateral surgery, 3/5 had identical LOAD and COI scores for both forelimbs, one had different scores for each limb, and one did not have numerical scores available. The COI and LOAD scores are graphically presented in Figure 5.

## 4 Discussion

This study aimed to report the owner-perceived long-term outcome and complications in a cohort of dogs with complex antebrachial deformities that underwent corrective ostectomy with the aid of patient-specific, 3D-printed osteotomy and reduction guides. We have found excellent long-term outcomes with full function in 19/20 limbs and acceptable function in the remaining limb. Two distinct types of major complications were seen: surgical site infection and implant-related soft tissue irritation, with an overall major complication rate of 35%. All complications were treated successfully with partial or complete implant removal.

Accurate assessment and surgical correction of canine antebrachial deformities is challenging. CAD has been used with CORA principles for preoperative planning (13, 14, 18–20); however, CORA relies on two-dimensional derived reference values, which are inaccurate when applied to multiplanar deformities (12, 25). More recently, CAD assessment with subjective segment orientation has been reported with good results (15–17). Proposed advantages of this technique include improved visualization of the deformity and restored alignment in all three planes, as well as the withdrawal of the requirement for reference values for joint orientation angles. The accuracy of CAD with subjective segment orientation has been demonstrated to be high in a recent study using vector-based methodology (12). It has also been investigated in two recent retrospective clinical studies with positive results (16, 21). The accuracy of 3D-printed guides has been extensively evaluated in various applications, including corrective ostectomy, fracture fixation, screw insertion in challenging anatomic locations, maxillectomy and brain surgery (16, 22, 23, 26–30). All dogs in this study had complex, non-compensated bi-apical or multiapical deformities, and we selected a CAD method with subjective segment orientation due to the proposed benefits.

This study reports a larger number of dogs compared to previous studies. The perioperative, short- and mid-term outcomes at the veterinary follow-up were acceptable function for the majority of the limbs (18/20). These outcomes compare favorably to the largest and most recent cohort of dogs that underwent corrective antebrachial ostectomies with the use of custom-made 3D-printed guides (16). Of the 11 dogs (13 limbs) operated on in that study, 2 guides were abandoned due to soft tissue tension, two cases presented with implant sensitivity, one case suffered intraoperative transection of the extensor carpi radialis tendon, and one case suffered fracture of the proximal radial segment 2 days post-surgery. No guides were abandoned in our study, and no intraoperative complications occurred.

The long-term outcome at our owner follow-up was full function in 19/20 limbs and acceptable function in the remaining limb, with a mean time of 23 months. Case 10, with a late recurrence of intermittent lameness in one of the affected limbs, had the most severe deformities in this cohort with bilateral radial head subluxation (Table 1; Figure 2). Due to the severity of the deformities and degree of elbow incongruence, this dog was anticipated to suffer from persistent low-grade lameness. In two previous studies using the same methodology (CAD subjective segment orientation and patient specific 3D-printed guides), 4/4 dogs showed slight improvement in COI with elimination of the previous carpal buckling over in a median follow-up time of 19 months (15) and 10/10 dogs in which guides were successfully utilized showed significant improvement in limb function scores in a median follow up time of 10 months (16). In another study using a similar methodology (no reduction guides were used), 6/6 limbs were reported to have improved COI scores, with full function in 3/6 limbs and acceptable function in the remaining 3/6 limbs, at a median follow-up time of 70 months (19). A direct comparison of outcomes across studies on antebrachial deformity correction in dogs is difficult due to the non-uniform methods of outcome assessment and the subjective interpretation by authors and readers. For consistency, we have not compared our long-term outcomes with those of case reports or older studies using CORA-based planning, freehand ostectomies and/or various methods of internal or external fixation.

The mean LOAD score was 4.4, with no individual score higher than 10. These values fall within the mild category (0–10) of LOAD score results, indicating mild arthropathy (31). Similarly, the mean COI score was 3.5, with no individual score higher than 10. The minimal clinically important differences for LOAD and COI have been estimated at 4 and 14, respectively, in dogs treated for cranial cruciate ligament disease in a recent study (32). This finding is also suggestive that dogs with minimal mobility issues can have LOAD and particularly COI scores above zero. Although LOAD and COI questionnaires were initially validated for use in dogs with osteoarthritis, they have been shown to correlate well with other validated clinical metrology instruments for mobility impairments in dogs (33, 34), and they have been used in previous studies to assess outcomes after antebrachial deformity correction in dogs (15, 19). The owner follow-up time reported here is longer than in the majority of previous studies [median 10 months (6–16 months) (16); median 19 months (12–32 months) (15)].

The incidence of implant-associated infection in our study was 3/20 (15%), which is higher than expected for clean surgery. However, this result should be interpreted cautiously due to the relatively small population of the study. One case presented with a late SSI (8 months postoperation), which would have been missed in a study with a shorter follow-up time. Surgical site infection remains one of the most important complications in small animal orthopedic surgery, and its prevalence is reported to be 3–17% (35–39). Previously identified risk factors for SSI include increased surgery time (35, 37), increased anesthesia time (40), an increased number of persons in the operating room (35) and the presence of an implant (36). One case of SSI had a longer surgical time than usual (2 h 20 min) and was performed by a relatively inexperienced surgeon. Other factors that may have contributed to the SSI rate are the extensive disruption of the soft tissues required to expose the radius sufficiently for the guides’ footprint and the production of a significant amount of debris during cutting and removing the polymerized resin material. A potential risk associated with SSI is osteomyelitis; however, no evidence of osteomyelitis was detected in radiographs obtained before or after implant removal or during explanation surgery. Bone union developed uneventfully in all cases, and there was no recurrence of clinical signs in any of these cases. Prevention of postoperative infection can be achieved through the reduction of anesthetic and surgical time as surgical experience with the described techniques increases, and by minimizing debris contamination of the surgical site through the use of damped gauze swabs to retain it outside the surgical field. All dogs included in this study received pre- and intraoperative antimicrobial prophylaxis as part of a standard protocol; however, no dogs received postoperative antibiotics. A prospective study of 100 consecutive clean orthopedic implant procedures found a reduction in the risk of SSI by ~84% with postoperative antimicrobials (38). However, a more recent systematic review on the efficacy of postoperative antibiotics after tibial plateau leveling osteotomy in dogs found little evidence to support their use (41). Given the high infection rate in our sample, further prospective research on the use of postoperative antimicrobials for antebrachial deformity in dogs using patient-specific 3D-printed guides may be useful.

The most common complication in our study was implant-related soft tissue irritation (4/20 limbs, 20%). Three of these limbs had double radial ostectomies and double ulnar osteotomies, and they were also the only cases that required that combination of cuts, reflecting the degree of needed antebrachial realignment. The fourth limb had a single radial osteotomy and a double ulnar osteotomy. Additionally, in some cases, the very distal location of the radial osteotomy meant that the bone plates were placed directly under the extensor tendons. Cranial plating of the intact canine radius has resulted in marked histologic changes in the extensor tendons and surrounding soft tissues, as well as a significant decrease in carpal flexion in an experimental study (42). In our cases, the radius was osteotomized in a single or double level, realigned and fixed with a system of 1–3 plates. This process presumably led to marked mechanical changes in the tendons and surrounding soft tissues, resulting in lameness and decreased carpal function. The clinical signs resolved or improved significantly in all cases following partial or complete removal of the implants. Prevention or mitigation of this complication may not be possible; a variety of biomechanically appropriate locking plates with relatively low profiles were utilized, with none of them apparently associated with increased incidence of soft tissue irritation necessitating plate removal (Table 2). Based on these findings, it may be recommended that owners of dogs undergoing these procedures should be advised that implant removal will be needed in the future.

Postoperative complications associated with antebrachial deformity correction with internal fixation in dogs include infection, self-mutilation over the radial plate (11), screw loosening, suboptimal torsional correction of the radius (19), osteopenia of the radius and decreased carpal range of flexion (43). Infection has been reported in 2/6 limbs in one study where no guides were used (11), but not in any of the other studies or case reports (13–16, 19–21, 43). Decreased carpal range of flexion was the most common complication (8/18 limbs) in a study describing T-plate fixation of distal radial closing wedge ostectomies (43) and appears to be similar to implant-related soft tissue irritation described in this study. While we found that implant removal significantly improved lameness in our dogs, plate removal was not deemed necessary in any of the dogs of that study and was not performed.

The main limitation of this study is its retrospective nature. However, the diagnostic investigation, preoperative planning, surgery, and postoperative care were largely uniform among the dogs in the study, and the surgeon was the same in the majority of procedures. The long-term follow-up was owner-assessed, which may introduce subjective bias. It would have been preferable to compare the LOAD and COI scores at the owner follow-up with those obtained preoperatively, but this was not possible due to the retrospective nature of the study. A prospective future study utilizing real-time preoperative and follow-up LOAD and COI scores or objective gait analysis would add significant information about the outcome of these surgeries. Finally, the lack of a control group treated with conventional CORA planning and/or non-guided ostectomies precludes a direct comparison with the methodology described here, and a prospective study would be needed for this purpose.

In conclusion, our study provides evidence that the acute correction of canine antebrachial deformities, assessed by subjective segment orientation and performed with the aid of patient-specific, 3D-printed osteotomy and reduction guides, can yield excellent long-term functional outcomes. Two distinct types of postoperative complications were found that can be both treated successfully with the removal of the implants after bone union.

## Data Availability

The raw data supporting the conclusions of this article will be made available by the authors, without undue reservation.
